# Investigating the shared genetic architecture between COVID-19 and obesity: a large-scale genome wide cross-trait analysis

**DOI:** 10.3389/fendo.2024.1325939

**Published:** 2024-01-30

**Authors:** Yanjing Chen, Chunhua Fan, Jun Liu

**Affiliations:** ^1^ Department of Radiology, Second Xiangya Hospital, Central South University, Changsha, Hunan, China; ^2^ Clinical Research Center for Medical Imaging in Hunan Province, Changsha, Hunan, China

**Keywords:** GWAS, COVID-19, obesity, genetic overlap, pleiotropy, genetic correlation

## Abstract

Observational studies have reported high comorbidity between obesity and severe COVID-19. The aim of this study is to explore whether genetic factors are involved in the co-occurrence of the two traits. Based on the available genome-wide association studies (GWAS) summary statistics, we explored the genetic correlation and performed cross-trait meta-analysis (CPASSOC) and colocalization analysis (COLOC) to detect pleiotropic single nucleotide polymorphisms (SNPs). At the genetic level, we obtained genes detected by Functional mapping and annotation (FUMA) and the Multi-marker Analysis of GenoMic Annotation (MAGMA). Potential functional genes were further investigated by summary-data-based Mendelian randomization (SMR). Finally, the casualty was identiied using the latent causal variable model (LCV). A significant positive genetic correlation was revealed between obesity and COVID-19. We found 331 shared genetic SNPs by CPASSOC and 13 shared risk loci by COLOC. At the genetic level, We obtained 3546 pleiotropic genes, among which 107 genes were found to be significantly expressed by SMR. Lastly, we observed these genes were mainly enriched in immune pathways and signaling transduction. These indings could provide new insights into the etiology of comorbidity and have implications for future therapeutic trial.

## 1 Introductions

The 2019 coronavirus disease pandemic (COVID-19) is a highly contagious disease caused by the Severe Acute Respiratory Syndrome Coronavirus 2 (SARS-CoV-2). Since January 2020, nearly 600 million people have been infected worldwide with the SARS-CoV-2 virus (1). Due to the widespread presence of the ACE2 receptor, which serves as the site of infection for the coronavirus, it has the potential to cause damage to various systems throughout the body. Obesity, as a public health epidemic, affects over 650 million adults globally and 124 million children and adolescents (2). Considering the high infectiousness and serious adverse outcomes of COVID-19 and the high prevalence of obesity (3), an in-depth exploration of the relationship between the two could help to develop effective policies and personalized treatments to control the spread of the epidemic and save social healthcare expenditures.

Previous studies have found that COVID-19 syndrome exacerbates metabolic dysfunction (4), including diabetes mellitus (5), vertebral fractures (6), and obesity. A study showed that 90% of SARS-CoV-2-infected patients with respiratory failure had a BMI higher than 25 kg/m2 (7). From an immunological point of view, excess energy in the diet accumulates in white adipose tissue, where the immune cells affect the overall balance through metabolism. For example, hypertrophied adipocytes recruit polarized macrophages that produce excessive amounts of inflammatory cytokines such as IL-6, TNF-α, and IL-1 (8) and blood levels of the proinflammatory adipokine leptin are elevated, while expression of the anti-inflammatory ACE2 receptor is reduced in lung epithelial cells (9). This imbalance significantly impairs the efficiency of the innate immune response. In addition, the sequelae of post COVID-19, also known as long COVID-19, has received more and more attention as the number of people found to be gradually increasing and affecting a number of systems and the mechanism of occurrence is not known (10), while some studies have found that exercise can be effective in alleviating post-COVID-19 syndrome and improving the physical strength and respiratory function of patients with COVID-19 (11). Using Mendelian randomization (MR), Xiong et al. found that physical activity and recreational sedentary behavior were associated with COVID severity and hospitalization rates (12).Therefore, obesity as the development of a variety of diseases and prognostic risk factors, and to understand its relationship with the COVID-19 is of profound significance. Since most of the past studies have been retrospective or observational, they are prone to many confounding factors that can bias the results, and as obesity is a highly hereditary disease, the aim of this study is to examine whether it is an intrinsic cause of neo coronary pneumonia disease from a genetic perspective and to reveal the anatomical and physiopathological mechanisms that are dependent on this.

Understanding the genetic architecture of both may not only explain the higher risk or worse prognosis of COVID-19 in obese individuals compared to normal individuals, but also provide insight into the pathogenesis of SARS-CoV-2, which help effectively manage obesity in the context of COVID-19. This study employed genome-wide cross-trait analysis to identify overlapping and distinct genetic architectures, thereby offering novel insights into disease mechanisms.

## 2 Materials and methods

**Figure 1:**
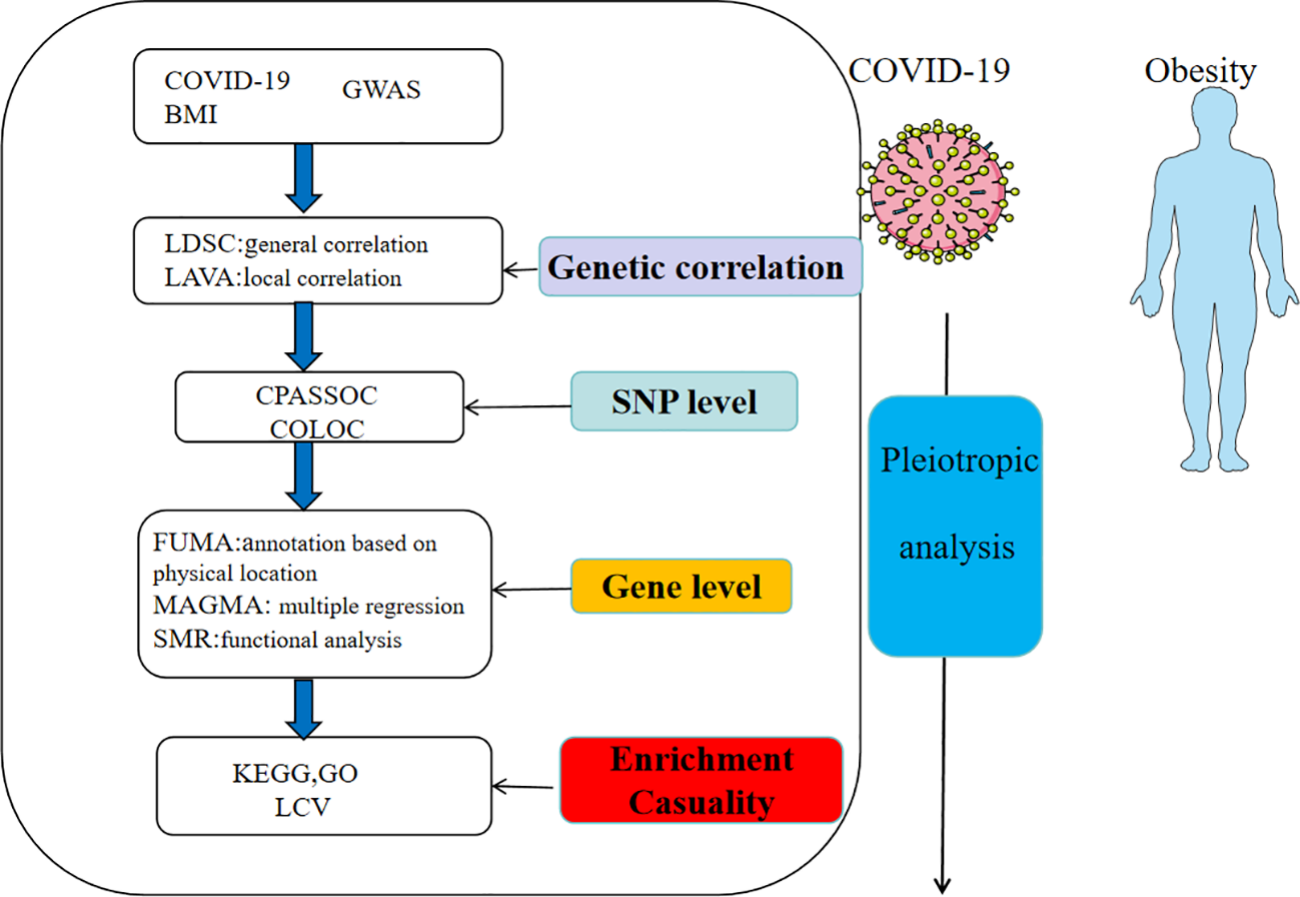
Overview of research of shared genetic architecture between COVID-19 and obesity.

The flowchart was shown in **Figure 1** and the figure were from smart(https://smart.servier.com/). The GWAS for COVID-19 was derived from the COVID-19 Host Genetics Initiative (https://www.covid19hg.org/), an international consortium aimed to discover genetic variants associated with susceptibility and severity of COVID-19. The GWAS of COVID-19 from the European population was obtained from the COVID-19 HGI GWAS round 7, including hospitalized COVID-19, critical COVID-19 and SARS-CoV-2 infection. The SNPs linked to BMI were acquired from the GIANT consortium, a meta-analysis including 2.4 million SNPs (13). To standardize the data, we first filtered out SNPs that was not present in the 1000 Genomes European population. Then we excluded SNPs without rsIDs or with duplicate rsIDs. At last, we addressed missing data by filling it in and mapped the chromosomal positions to the hg19 reference genome.

### 2.1 General genetic correlation analysis

Because heritability (h2) is distributed over thousands of variants with small effects, it is not sufficient to focus only on SNPs that differ or are significant between or within traits. In order to measure the average sharing of genetic effect across the entire genome between obesity and COVID-19, we used the linkage disequilibrium score regression (LDSC) to estimate h2 (14) and genetic correlation (rg) (15) based on the summary GWAS statistic. With reference data obtained from the third phase of the European 1000 Genomes (1KG) project, LDSC can integrate the associated evidence for multiple traits of interest (continuous and dichotomous) from one or more studies. The LD Score regression intercept was employed to estimate a more powerful and accurate correction factor compared to genomic control.

### 2.2 Local genetic correlation analysis

Since traditional global approaches only consider the average rg across the genome, they may fail to detect scenarios where the overlapped information is confined to specific regions or has opposing directions at different loci. We used LAVA (Local Analysis of Variant Association) which can detect shared genetic association regions between phenotypes by utilizing local genetic regions (16). And the pairwise local rg tests on 2,495 genomic loci (the entire genome) applying multivariate genetic association analysis can provide more complex and conditional genetic relationships.

### 2.3 Cross-trait meta-analysis

For the purpose of identifying the risk SNPs associated with joint phenotypes (COVID-19 and obesity), we implemented Cross Phenotype Association (CPASSOC), which allows meta-analysis of continuous traits based on the GWAS. There are two statistical methods, SHom and SHet. SHom is an extension of the linear combination of univariate test statistics, allowing sample size to be used as weights and its statistical power is diminished when there is between-study heterogeneity. Thus, we utilized SHet for analysis which can sustain statistical power even in the presence of heterogeneity by assigning greater weights. Since SNP often cannot directly determine causal variants due to the influence of linkage imbalances, the Functional mapping and annotation (FUMA) was utilized (17) to provide annotation information for SNPs associated with functional categories, especially regarding non-coding regions or intergenic regions. Among the provided information, CADD scores above 12.37 indicate potential detrimental effects on protein outcomes and the scores from RegulomeDB offer valuable insight into the regulatory functionality of SNPs by considering their association with expression quantitative trait loci and chromatin marks.

### 2.4 Colocalization analysis

The meta-analysis of various traits resulted in the inclusion of genetic loci associated with individual traits. Subsequently, we employed the colocalization method (COLOC) (18) with the aim of investigating whether the same genetic variation in the loci is responsible for both traits. This algorithm is using a Bayesian algorithm to calculate posterior probabilities for five exclusive hypotheses related to the sharing of causal variants in a genomic region. These hypotheses include H0 (no association), H1 or H2 (association with one specific trait), H3 (association with both traits, involving two distinct SNPs), and H4 (association with both traits, involving one shared SNP). A locus is considered colocalized if PPH4 or PPH3 is greater than 0.7. We utilized the R package “coloc” in Rstudio software to extract summary statistics for variants within 5 Mb of the topSNP at each shared locus after annotating in FUMA.

### 2.5 Multi-marker analysis of GenoMic annotation

Gene and gene-set analysis have been suggested as potentially more powerful alternatives to the typical single-SNP analyses performed in GWAS. The FUMA can provide annotation of SNPs to genes based on physical location. In addition, we used MAGMA (Multi-marker Analysis of GenoMic Annotation) (19) to obtain genes or sets of genes significantly associated with traits. It is a fast and flexible tool which uses a multiple regression approach to properly incorporate LD between markers. We compared the gene sets generated by MAGMA with the gene sets annotated based on physical location. After applying the Bonferroni correction, the resulting genes represented the final set of pleiotropic genes identified at the gene level. Additionally, we performed GTEx tissue enrichment analysis using MAGMA and the 54 tissue types from GTEx (v.8) to determine the specific tissues associated with the shared genes. To address multiple testing, we adopted the Benjamin-Hochberg procedure.

### 2.6 Summary-data-based Mendelian randomization

We used Summary-data-based Mendelian randomization (SMR) to identify putative functional genes underlying statistical associations for obesity and COVID-19. SMR can integrate the GWAS and eQTL to investigate the expression of the pleiotropic genes in mRNA level, which was under the MR framework to test for an link related to gene expression and a target phenotype (20). The source of eQTL were based on 2 different reference panels, Genotype-Tissue Expression project (21) (GTEx) and the Encyclopedia of DNA Elements project (22) (ENCODE). The SMR can be also used to perform the heterogeneity in dependent instruments (HEIDI) test to evaluate the existence of linkage in the observed association. Significant common shared functional genes between COVID-19 and obesity were defined passed the threshold (p<0.05) and HEIDI-outlier test (p > 0.01) in SMR analyses of both traits.

### 2.7 Enrichment analysis

To gain a better understanding of the biological implications of the final pleiotropic genes identified from the overlapped genes detected by MAGMA and the result of annotation, we performed an enrichment analysis of these genes in terms of Gene Ontology (GO) biological processes (23) and Kyoto Encyclopedia of Genes and Genomes (KEGG) (24) pathways using the “clusterProfiler” R package. These two analysis can reveal the enriched biological functions and metabolic pathways in a gene set. The GO analysis annotates genes to biological processes (BP), molecular functions (MF), and cellular components (CC) in a hierarchically structured manner.

### 2.8 Latent causal variable analysis

At last, to further study whether the genetic correlated relationship between the COVID-19 and BMI have the casual component, The latent causal variable (LCV) model (25) was used in this study, which is mediated by a latent variable that causally impacts each trait. Compared to MR, it can overcome the heterogeneity of instrumental variables. We introduced the concept of genetic causality proportion (GCP) to measure the degree of partial causality and quantify the impact of BMI on COVID-19. The GCP scale spans from 0 to 1, representing the absence of partial genetic causality and the presence of full genetic causality, respectively.

## 3 Results

### 3.1 Genetic correlations

The heritability of BMI was 0.2116, and the heritability of critical COVID-19 was 0.0062, which was the highest of the three pair traits. The general genetic correlation between obesity and COVID-19 was positive. We identified that the rg was 0.363 between BMI and hospitalized COVID-19, and the P value was 8.91E-15. There was also a significant rg (rg=0.3451, P=1.54E-08) between BMI and critical COVID-19. A link between COVID-19 and BMI (rg=0.0722, P=8E-04) could be found. The most overlap rate was only 0.018 between the critical COVID-19 and BMI (**Table 1**). In LAVA, we all found the seven local relationships between hospitalized COVID-19 and BMI. Among them, the most significant locus was located at chr: bp 1:77895395-79065286, with a P-value of 9.84E-09. The second significant locus was found at chr: bp 7:13676748-15013694, with a P-value of 9.32E-07. The locus located in chr 19:55182974-55714085 was observed to be shared between COVID-19 and BMI. We did not observe any additional region that showed a significant local genetic correlation between critical COVID-19 and BMI (**Table 2**).

**Table 1:** The source of GWAS and genome-wide genetic correlation between COVID-19 and BMI using LDSC.

The detailed information				LDSC			
Diseases	N_cases	N_control	Ancestry	h2	rg	P	inter
BMI	387649	0	EUR	0.2116			
Hospitalized COVID-19	32519	2062805	EUR	0.0036	0.3639	8.91E-15	0.0244
Critical COVID-19	13769	1072442	EUR	0.0062	0.3451	1.54E-08	0.0183
COVID-19	122616	2475240	EUR	0.0019	0.0722	0.0008	0.0238

h2, heritability; rg, genetic correlation; inter, quantity overlap of population, inter represents the overlap of two samples.

**Table 2:** The local genetic correlation between COVID-19 and BMI in 2495 loci by LAVA.

Loci	CHR	START	STOP	PHE1	H2	P1	PHE2	H2	P2	P
67	1	77895395	79065286	Hospitalized COVID-19	3.80E-05	2.21E-06	BMI	4.84E-04	1.11E-43	9.84E-09
1113	7	13676748	15013694	Hospitalized COVID-19	7.45E-05	3.24E-09	BMI	4.74E-04	2.38E-30	9.32E-07
2201	17	34474612	36116884	Hospitalized COVID-19	4.66E-05	8.49E-07	BMI	4.56E-04	1.26E-32	1.68E-06
1197	7	113339387	115321301	Hospitalized COVID-19	2.53E-05	1.13E-03	BMI	5.13E-04	1.30E-42	4.27E-06
2311	19	3893910	4741718	Hospitalized COVID-19	1.78E-04	8.97E-47	BMI	3.72E-04	2.68E-26	1.05E-05
692	4	102544804	104384534	Hospitalized COVID-19	3.27E-05	1.36E-04	BMI	5.06E-04	1.58E-40	1.38E-05
56	1	65894185	66778015	Hospitalized COVID-19	1.45E-05	1.39E-02	BMI	1.91E-04	6.98E-14	1.91E-05
2364	19	55182974	55714085	COVID-19	1.22E-04	3.89E-40	BMI	9.30E-05	3.98E-05	3.65E-07

CHR, chromosomes; START, genomic starting point; STOP, genomic ending point; H2, heritability; rg,genetic correlation; PHE, phenotype, P1 and P2 represent the heritability of the trait in the region.

### 3.2 Shared loci between obesity and COVID-19

Cross-trait meta-analysis can improve the test efficacy by integrating the GWAS of two traits using meta-analysis ideas, which can help us discover potential new pleiotropic SNPs. We used the CPASSOC method and established a threshold of P < 5 × 10−8 for meta-analysis and P < 5 × 10−5 for the single trait analysis. In our study, a total of 331 SNP were identified between COVID-19 and BMI (**Supplementary Table 1**), among which 39 SNP were linked to COVID-19, 207 SNP were linked to hospitalized COVID-19, and 85 SNP were related to critical COVID-19. The most significant SNP between critical COVID-19 and BMI was the rs13107325 (P_critical COVID-19 = _1.71E-05, P_BMI_=1.10E-47, P_CPASSOC_=2.06E-48). The SNP rs2088518 showed a most significant association (P_CPASSOC_=8.60E-28) with COVID-19 (P = 4.17E-05) and BMI (P = 3.10E-28). The rs13107325 (P_CPASSOC_=1.67E-48) was the most significant locus shared between hospitalized COVID-19 (P=1.10E-47) and BMI (P=1.33E-07). We identified 17 novel SNP (5E-8<P_single trait_<1E-5 and P_CPASSOC_<5E-8) between hospitalized COVID-19 and BMI, 3 novel SNP between COVID-19 and BMI, and 2 novel SNP between critical COVID-19 and BMI (**Supplementary Table 2**).

After physically annotating the polytropic SNPs obtained from the above results in the FUMA, we can get the corresponding risk loci. Further colocalization analysis identified that there were 13 loci with a PPH4 value exceeding 70% (**Table 3**), 7 loci were associated with critical COVID-19, 5 were connected with COVID-19, and 1 was related to hospitalized COVID-19. The corresponding SNP, rs1813006, shared between critical COVID-19 and BMI, was consistent with critical COVID-19 (PPH4 = 0.990) and was the most significant locus (PPH4 = 0.999). The locus between hospitalized COVID-19 and BMI (PPH4 = 0.998) was located at chr: bp 19:4069119. It mapped to five genes (PPP1CB, SPDYA, TRMT61B, WDR43, FAM179A).

**Table 3:** Results from colocalization analysis for each pleiotropic locus identified from CPASSOC.

TopSNP	Chromosome	Position	Genomiclocus	A1	A2	COVID-19			BMI		*P*_CPASSOC_	PPH3	PPH4
						Trait	Beta	*P*-value	Beta	*P*-value			
rs7254272	19	4069119	58	A	G	Hospitalized COVID-19	-0.07	1.16E-07	-0.02	5.90E-17	8.62E-17	0.002	0.998
rs1813006	4	103001649	187	T	G	COVID-19	0.04	6.76E-06	0.05	4.40E-35	2.25E-34	0	1
rs8070454	17	38160754	596	T	C	COVID-19	-0.02	1.65E-05	-0.01	4.20E-09	3.23E-08	0.053	0.88
rs7788008	7	112972483	322	A	G	COVID-19	-0.01	0.084829	-0.02	1.10E-19	1.05E-19	0.022	0.821
rs6901756	6	41825590	261	C	T	COVID-19	-0.03	5.65E-05	-0.01	2.90E-09	2.07E-08	0.004	0.818
rs11085735	19	10602180	635	A	C	COVID-19	-0.04	1.22E-06	0.01	3.30E-05	2.60E-08	0.042	0.729
rs1813006	4	103001649	187	T	G	Critical COVID-19	0.11	8.99E-05	0.05	4.40E-35	1.58E-34	0.001	0.99
rs8070454	17	38160754	598	T	C	Critical COVID-19	-0.07	7.31E-06	-0.01	4.20E-09	3.23E-08	0.031	0.956
rs17391694	1	78623626	25	T	C	Critical COVID-19	0.02	8.79E-05	0.03	7.50E-38	1.70E-36	0.02	0.937
rs1075901	17	15943910	593	T	C	Critical COVID-19	-0.05	0.00011458	-0.01	1.20E-13	1.63E-13	0.05	0.904
rs17484848	2	111900560	81	C	T	Critical COVID-19	-0.09	0.000122	0.01	2.10E-07	7.80E-10	0.005	0.87
rs11085735	19	10602180	639	A	C	Critical COVID-19	0.2	7.69E-13	0.01	3.30E-05	7.64E-15	0.04	0.851
rs7560871	2	145616899	86	A	G	Critical COVID-19	0.09	0.0006997	0.02	9.60E-11	6.10E-10	0.079	0.816

PHH3/PHH4:The percentages of H3 (association with both traits, involving two distinct SNPs) and H4 (association with both traits, involving one shared SNP).

### 3.3 Candidate pleiotropic genes and tissue specificity

**Figure 2:**
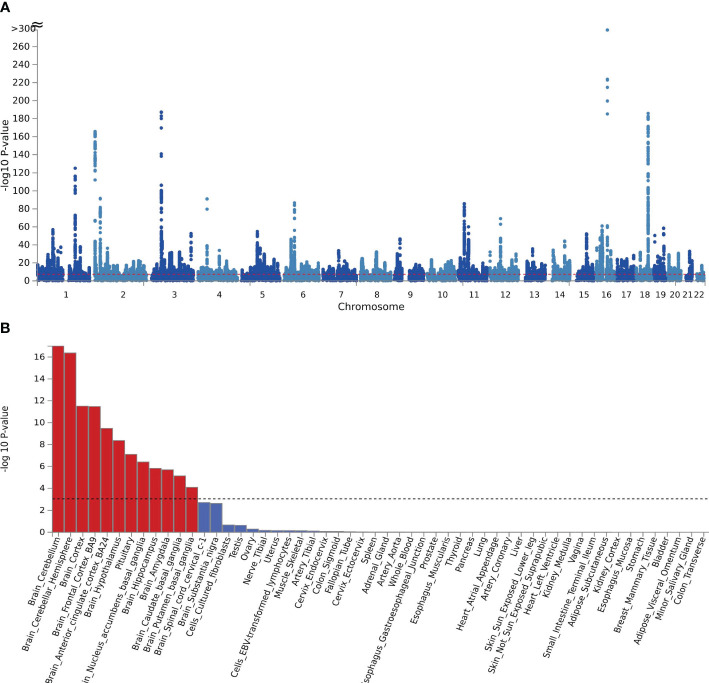
**(A)** The manhattan plot of the SNP-based test based on results of CPASSOC of crirical COVID-19; **(B)** the result of tissue enrichment in GTEx 8 of shared genes between critical COVID-19 and BMI, the color red represents the significant tissue, the color blue represents the tissue did not reach threshold.

MAGMA was analyzed based on the results of CPASSOC, and it found that 5162 genes overlapped and were mapped by loci physically annotated by FUMA (**Supplementary Table 3**). Among these genes, there were 273 genes associated with hospitalized COVID-19, 2433 genes associated with COVID-19, and 2456 genes associated with critical COVID-19. After applying the Bonferroni correction, MAGMA identified 3546 significant pleiotropic genes, out of which 152 genes were detected in 2 or more trait pairs. The most common genes were shared between COVID-19 and critical COVID-19 (**Supplementary Table 4**). We observed significant enrichment for BMI and COVID-19 in brain tissues after correction in every form of COVID-19. The results were identified in 10 brain regions, including the brain cerebellum(P=1.66E-29), the brain cerebellar hemisphere (P=2.98E-15) in COVID-19, and the result of critical COVID-19 (Pbrain cerebellum = 1.02E-17, Pbrain cerebellar hemisphere=4.28E-17).The enrichment between hospitalized COVID-19 and BMI mainly enriched in the brain cortex (P=4.55E-07) and brain frontal cortex BA9 (P=3.05E-06) (**Supplementary Table 5**, **Figure 2B**). The further functional gene analysis using SMR found 20 significant genes between hospitalized COVID-19 and BMI, 39 genes significant genes between critical COVID-19 and BMI, and 48 genes between COVID-19 and BMI (**Supplementary Table 6**).

Combined with the above result, we show seven common genes significantly expressed in all analytic methods at both SNP and genetic levels (**Table 4**). Among them, ADORA2B was on the chr17 and was significantly expressed in blood, lung, brain frontal cortex BA9, and brain cerebellar hemisphere. The most significant tissue was on the lung (P_critical COVID-19 = _1.018E-03, P_BMI_=7.37E-07), and the PHEIDI was all above 0.01. Apart from this, ZSWIM7 was the most common gene seen between BMI and critical COVID-19, which was found in 12 tissues. The expression in the brain cortex was most significant (P_BMI_= 1.12E-04, P_COVID-19 = _3.09E-09). There was only one significant gene detected by COLOC and SMR between COVID-19 and BMI, which was CCND3. It was on the CHR 6 and significantly expressed in whole blood (P_COVID-19 = _0.028, P_BMI_=2.30E-05). There were no functional genes shared between hospitalized COVID-19 and BMI.

**Table 4:** The significant functional genes detected in both COVID-19 and BMI.

Gene	Tissue	Trait	Genomic Locus	CHR	START	STOP	NSNPS
ZSWIM7	Brain caudate basal ganglia, Brain cerebellar hemisphere, Brain cerebellum, Brain cortex, Brain frontal cortex_BA9, Brain hypothalamus,Brain nucleus accumbens basal ganglia,Brain putamen basal ganglia,Lung,ENCODE,Amygdala,Brain anterior cingulate cortex	Critical COVID-19	593	17	15879874	15903031	13
ADORA2B	Whole blood,Lung,Brain frontal cortex_BA9, ENCODE, Brain cerebellar hemisphere	Critical COVID-19	593	17	15848231	15879060	15
TTC19	Brain nucleus accumbens Basal ganglia,Lung,Brain putamen basal ganglia,Brain caudate basal ganglia,Whole blood	Critical COVID-19	593	17	15897694	15953329	34
GSDMA	Lung	Critical COVID-19	598	17	38119226	38134019	12
DNM2	ENCODE	Critical COVID-19	639	19	10823755	10949164	54
SMARCA4	ENCODE	Critical COVID-19	639	19	11066598	11181071	55
ZZZ3	ENCODE	Critical COVID-19	25	1	78023101	78154104	64
CCND3	Lung,ENCODE	COVID-19	261	6	41897671	42023095	73

Tissue: the souce of eqtl, ENCODE, Encyclopedia of DNA Elements project; CHR, chromosomes; START, genomic starting point; STOP, genomic ending point; NSNPS, number of SNPs within the gene.

### 3.4 Biological mechanisms shared between COVID-19 and BMI and causality

**Figure 3:**
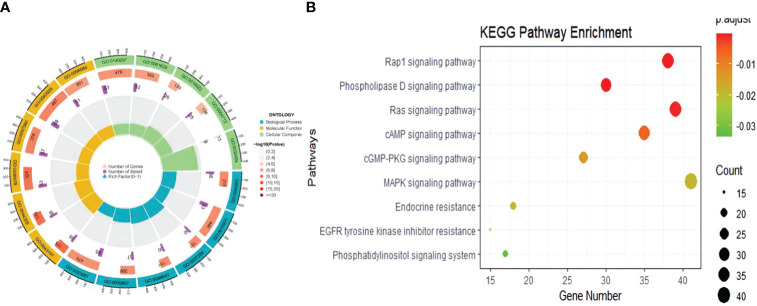
The results of enrichment of the shared genetic architecture between COVID-19 and obesity. **(A)** The circle plot of GO result, **(B)** The bubble plot of KEGG analysis.

In the biological process of GO analysis, we identified pleiotropic genes that were commonly associated with both COVID-19 and BMI. Specifically, these genes were significantly enriched in the regulation of synapse structure or activity (P=4.48E-07, Padjust=6.27E-04), which was consistent with their critical role in COVID-19 (P=1.42E-06, Padjust=7.23E-04). At the molecular function level, our results indicated that the genes between critical COVID-19 (P=5.84E-07, Padjust=5.79E-04) or COVID-19 (P=1.01E-06, Padjust=1.00E-03) and BMI are primarily involved in DNA-binding transcription factor binding. Furthermore, the enrichment of these genes at the cellular component level was relatively minimal (**Figure 3**, **Supplementary Table 7**).In the KEGG analysis, we found that the pleiotropic genes shared between hospitalized COVID-19 patients and BMI were predominantly enriched in the Phospholipase D signaling pathway (P=5.84E-07, Padjust=5.79E-05) and the Rap1 signaling pathway (P=3.26E-05, Padjust=1.1E-03), which was consistent with the findings on COVID-19 (**Supplementary Table 8**).

We cannot find the causal component in the genomic association between COVID-19 and BMI. The P value presented by LCV between COVID-19 and BMI was 0.78. It also indicated a lack of statistically significant association between hospitalized COVID-19 and BMI, with a p-value of 0.57. It yielded a p-value of 0.56 when assessing the association between critical COVID-19 and BMI.

## 4 Discussion

As one of the common risk factors for COVID- 19, a deep exploration of the genetic correlation and genetic pleiotropy between them can not only further our understanding of their interaction but also provide evidence that weight management facilitates the reduction of viral infections in the context of the COVID-19. Through LDSC, we discovered a positive genetic correlation between COVID- 19 and obesity regardless of the COVID- 19 type, which was consistent with previous epidemiological surveys. In order to reduce the bias brought by genetic structure from different locations on the overall correlation, we further conducted a local genetic correlation analysis. In the pleiotropy analysis, we found that 39 loci were linked to COVID- 19, 207 were linked to hospitalized COVID- 19, and 85 were related to critical COVID- 19. After validation with the COLOC algorithm, these loci yielded 13 loci with posterior probabilities more significant than 70% and were further mapped to 3545 genes through MAGMA analysis. Finally, combining the abovementioned filtering, we performed SMR analysis and obtained eight effective drug targets (PSMR<0.05, PHEIDI>0.01). This study provides a specific shared genetic structure for the comorbidity between obesity and COVID- 19, rather than just due to age and health status, etc.

The identification of biomarkers for COVID-19 may enable early detection of patients at risk of developing severe illness. Genetic factors can partially control the up- or down-regulation of amino acid pathways that play a role in the COVID-19 immune response (26). COVID-19 susceptibility or severity is determined by host genetic polymorphisms (27),in addition, Li et al. found that obesity plays an important role in the development of severe COVID-19 when studying the causal relationship between nonalcoholic fatty liver disease and COVID-19 (28). Hakonars ect found that obesity, rather than diabetes, is the crucial risk factor for hospitalization in COVID-19 patients (29).However, these studies only used MR to explore their causal relationship, without delving into the underlying genetic connections and quantifying the genetic architecture. We selected GWAS from two large-scale studies to elucidate the intersectionality that exists between the two traits through various genomic levels to ensure that the interpretation of COVID-19 and obesity is as adequate as possible. The diverse manifestation of COVID-19 can be attributed partly to the host’s genomic background. In contrast to the other two different types of COVID-19, the SMARCA4 is a particular gene we detected between critical COVID- 19 and BMI in all conducted methods, which encodes the ATP-dependent chromatin remodeling factor 4 (30). SMARCA4 has been identified as the second most significant gene after ACE2 for COVID-19 (31). A previous study found SMARCA4 was the essential mutation in children with ASD and widespread low-density lipoprotein-related lipidome derangements (32). Furthermore, this study revealed that the expression of the SMARCA4 gene in whole blood tissues influenced the development of both traits which is consistent with that SMARCA4-LDLR haplotypes were the determinant of plasma lipids, whose catalytic activity is necessary for ACE2 expression and viral susceptibility (33). Weight control may be the most crucial modifiable risk factor for preventing the development of severe COVID-19.

We identified some genes reported in previous studies to be associated with COVID-19 and obesity. The ADORA2A encodes the adenosine A2A receptor which regulates cellular signaling and biological functions through adenosine binding (34). Adenosine is known as an anti-inflammatory and healing molecule of the purinergic system. Selective agonists of A2AR may have significant potential to reduce the fatality of COVID-19 (35, 36). Studies have found that in terms of leukocyte gene expression, COVID-19 patients show upregulation of genes including (37) P2RX1, P2RY12, PANX1, ADORA2B, NLPR3, and F3 (38). Meanwhile, A2A receptors play a crucial pathophysiological role in obesity and related diseases (39) by participating in inflammation, insulin resistance, fat generation, and thermogenesis (40, 41). Previous studies have found that the expression of A2AR in the adipose tissue of obese mice induced by a high-fat diet (HFD) significantly increased and mainly existed in adipose tissue macrophages (42). It found that adenosine-A2AR signaling could activate brown adipose tissue, which has an anti-obesity effect by inducing the browning of white adipose tissue (43). In conclusion, the dysfunction of adenosine-A2AR mediated by ADORA2A mutations may be a common cause of obesity and COVID-19 due to disrupted adenosine function. what is more, the CCND3 gene encodes the protein Cyclin D3, a key molecule in cell cycle regulation, which is preferentially expressed in adipose tissue, and its expression is strongly induced during the terminal stages of 3T3-L1 adipogenesis (44). Moreover, cyclin D3 was identified to disrupt the function of envelope and membrane proteins of SARS‐CoV‐2 by affecting spike trafficking and incorporating the E protein into the virions (45). The discovery of these genes and the pathologic processes involved can provide insights for our future studies of obesity in neocoronogenesis and long neocoronary syndromes.

In this study, we found that brain regions are involved in the co-occurrence of both traits using SMR and enrichment analysis. The regions of the brain and neurons help maintain energy balance and homeostasis by perceiving and processing various metabolic signals observed in the hypothalamus. Meanwhile, the peripheral inflammation caused by COVID-19 may have long-term consequences on the neurological system of recovery patients, such as neurodegenerative diseases like dementia (46) The excessive intake of saturated fatty acids can activate the innate immune system and impair adaptive immunity, leading to chronic inflammation and compromised host defense against viruses and these outcomes may be worsened by continued excessive fat intake. In addition, we have also found a high enrichment of pleiotropic genes in the pituitary gland. COVID-19 patients with pituitary dysfunction experience changes in multiple endocrine organs, tissues, and hormone substances, making them susceptible to diabetes, obesity, and fractures (47). This is related to the expression of ACE2 mRNA in the hypothalamus and pituitary cells (48), indicating the close relationship between the management of pituitary diseases in the context of COVID-19 and the occurrence and development of complications. We also identified some shared genes not found to be associated with either trait in previous studies which were worthy of future study.

This article has several advantages. Previous studies almost exclusively used Mendelian randomization to investigate the causal relationship between obesity and COVID-19, which can be influenced by horizontal pleiotropy and cannot identify the specific genetic architecture underlying the effects. We deeply explored the genetic overlap between them from SNP to functional levels using various post-GWAS methods. Additionally, enrichment analysis was employed to reveal the genetic factors regulating anatomical and physiological changes. In terms of causal relationships, we used LCV, a model that quantifies the causal portion compared to MR and is less susceptible to confounding variables. Our study has limitations as we only used a European sample due to the limited sample size of other racial groups. Addition of future GWAS data could further enhance our research results. Second, our research’s findings requires to be confirmed through basic experiment. Our work only concentrated on the SNP, gene, and mRNA levels, detailed research can be done at the protein level in the future.

## 5 Conclusions

This paper reveals in detail the genetic structure of the new crown and obesity and supports their intrinsic link, a finding that provides strong support for future decisions on weight management to reduce COVID-19 infection and development.

## Data availability statement

The original contributions presented in the study are included in the article/**Supplementary Material**. Further inquiries can be directed to the corresponding author.

## Author contributions

YC: Conceptualization, Data curation, Formal analysis, Writing – original draft. CF: Software, Supervision, Writing – original draft. JL: Writing – review & editing.
